# Measurement of Axial Strain of Geogrid by Optical Sensors

**DOI:** 10.3390/s21196404

**Published:** 2021-09-25

**Authors:** Marian Drusa, Ladislav Kais, Jozef Dubovan, Miroslav Markovic, Frantisek Bahleda, Martin Mecar

**Affiliations:** 1 Department of Geotechnics, Faculty of Civil Engineering, University of Žilina, Univerzitna 8215/1, 010 26 Žilina, Slovakia; 2 Tubau a. s. K Cintorínu 561/45, Bánová, 010 04 Žilina-Závodie, Slovakia; kais@tubau.sk; 3 Department of Multimedia and Information and Communication Technology, Faculty of Electrical Engineering and Information Technology, University of Žilina, Univerzitna 8215/1, 010 26 Žilina, Slovakia; jozef.dubovan@fel.uniza.sk (J.D.); miroslav.markovic@fel.uniza.sk (M.M.); 4 Department of Construction Materials and Bridges, Faculty of Civil Engineering, University of Žilina, Univerzitna 8215/1, 010 26 Žilina, Slovakia; frantisek.bahleda@uniza.sk; 5 Department of Railway Engineering and Track Management, Faculty of Civil Engineering, University of Žilina, Univerzitna 8215/1, 010 26 Žilina, Slovakia; martin.mecar@uniza.sk

**Keywords:** axial strain, geogrid, optical sensors, FBG sensors, physical model

## Abstract

In recent years, the technology of optical fibers has rapidly gained ground in many areas of science and industry, including the construction industry. In this article, the technology of optical fibers based on a fiber Bragg grating (FBG) was used to determine tensile forces acting in a basal reinforcement of a scaled down physical model, which included piled embankment and basal reinforcement. Installing FBG sensors on the geogrid made monitoring of axial strains possible, thus allowing determination of the behavior of the basal reinforcement of the piled embankment. On the basis of three tests performed on the physical model, numerical model calibration with the physical model was carried out using the software PLAXIS 3D Tunnel 2.4. The results showed accurate predictions, especially for the low and middle part of the measured deformations where the numerical analysis proposed a solution that can be considered as safe. Installing FBG sensors on biaxial geogrids was a bold idea that was not easy to implement. However, other possibilities have been successfully tested, such as high-frequency measurements of the response of reinforced soil structure under dynamic loading.

## 1. Introduction

In Central Europe, there is currently not enough experience with the implementation nor the monitoring of piled embankments and their basal reinforcements. Up until now, there have been only a few cases of this type of structure—one example being the modernized high-speed railway line between Bratislava–Zilina (Slovakia), in the section before and after the tunnel named Turkish hill in total length up to 2 km. The second realization was at the intermodal transport terminal at Nitra (Luzianky) next to the Jaguar Land Rover production plant in Slovakia. However, thorough geotechnical monitoring was not performed on the subject sections, which would without a doubt be useful not only for the contractors but also for the area of research and the subsequent optimization of design steps. Unfortunately, the ratio of realized constructions and constructions that are monitored is, in general, negligible. There are two reasons behind this, the first being the reluctance of contractors to invest in the monitoring, the second being the technical difficulties that are related to it. In this case, it is the basal reinforcement that poses significant issues. These issues lie in the unreliability of the monitoring equipment as well as problems concerning the installation and calibration of sensors along with other factors such as humidity and it being a generally mechanically-strained environment (clastic material, construction machinery, etc.).

**Figure 1:**
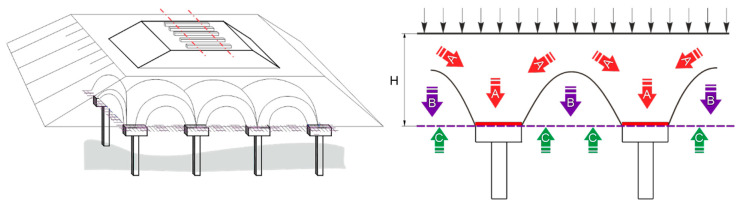
Scheme of piled embankment of railway track (**left**), load distribution on piled embankment in case of full arching (**right**).

During the monitoring of a construction such as piled embankment, it is necessary to monitor not only the distribution of the load in the piled embankment (Figure 1) but also the tensile forces acting in the basal reinforcement, which can cause axial strain of the reinforcement [1].

**Figure 2:**
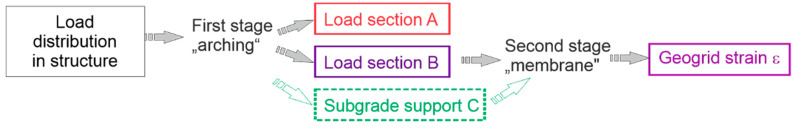
Distribution of the load in the piled embankment.

This is the reason why these parameters should not be underestimated when designing the basal reinforced piled embankment. Most of the analytical design models for basal reinforcements of piled embankments propose the design in two phases (Figure 2) [2,3,4]. The subject matter of this article was the second phase as this phase deals with determining the tensile forces in the reinforcement.

In most cases, the basal reinforcement consists of a geosynthetic reinforcement in one or multiple layers. Determining the tensile forces in the reinforcement is indirectly possible by using work diagrams and the axial strain of the reinforcement.

There have been multiple studies focused on determining the strain of the geogrid using various methods. For example, Bathurst et al. used tensiometric sensors with high strain to find a nonlinear correlation between local and global strain of the geogrid [5]. Van Eekelen et al. [6] used Bowden cables for measuring the axial strain of the geogrid, while Yang et al. used flexible sensors of displacement [7]. The strain of the geogrid can thus be determined using different methods. The above mentioned methods can be considered as conventional ways of determining the strain, however, every one of them has its advantages and disadvantages.

The disadvantage of the tensiometric sensors is that they cannot be reliably applied on most of the geosynthetic reinforcements. The problem lies in the inadequate or weak adhesion of the glue on the surface of the geosynthetic material because of either the surface structure or the insufficient area for gluing the sensor on the material. In the case that it is possible to glue the sensor to the reinforcement, another problem arises in the interpretation of the results where the influence of the glue can be enough to cause a deviation in results. Apart from their difficult installation, tensiometric sensors are also sensitive to sharp edges and they need to be thermally compensated.

Another way of measuring the strain is using Bowden cables, which are flexible hollow tubes that house a steel wire that can freely move. The end of the wire as well as the end of the rubber tube are firmly fixed to the geosynthetic reinforcement so that the difference between the length of the cable before and after the measurement can be seen. The main advantages of this measurement are the simplicity and lower financial demand [6]. On the other hand, the inaccuracy of such a measurement is one of the problems that Bowden cables (strain cables) can have in the small strain range of geogrid.

An unconventional way of measuring the strain of the geogrid was selected and that consisted of using the optical fibers such as in the case of Yijie Sun et al. [8]. In general, the technology of fiber optics offers the advantages of being a simple and accurate measurement. Other benefits include its resistance against external influences, such as humidity and the effects of the outer electromagnetic field (as the optical fiber is dielectric, there is no other interference by external sources) [9,10], which are interesting, especially in cases of long-term monitoring and complicated environmental conditions [8,11,12,13]. Without a doubt, one of the advantages is the ability to use fiber optics in the area of dynamic analysis where optical fibers are capable of measuring high frequencies [14,15,16]. On the other hand, higher investment costs and the lower range of the measurement of individual optical fibers are the main disadvantages as it is still a relatively new technology. There is also the question of the reliable application of the FBG sensor on the measured surface so that the data transfer of the strain is performed without any loss [17].

## 2. Optical Fibers with Bragg Gratings

### 2.1. History—Continuous Development

**Figure 3:**
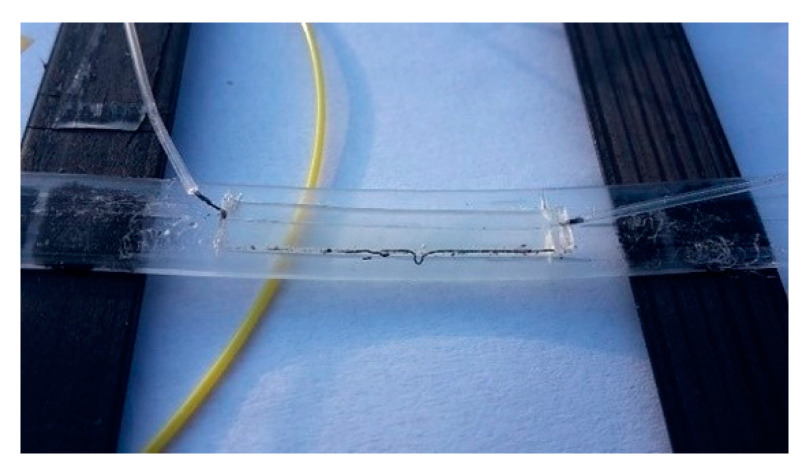
FBG sensor installed on the geogrid.

There is no doubt that the advantages of measurements with optical sensors find their place in many areas of applications, including geotechnical engineering and geotechnical monitoring of structures and the rock environment. Our measurements were made specifically using optical fibers with a Bragg grating (Figure 3). The physical foundations of these sensors were laid by W. H. Bragg and his son, W. L. Bragg, for which they were awarded the Nobel Prize in 1915. The first FBG sensor was made by Hill et al., a research team at the Communications Research Centre in Ottawa, Canada [18], who first discovered the phenomenon of photosensitivity in germanium-doped (Ge-doped) silica fiber.

A Bragg grating inside the optical fiber was first used in 1978 by the irradiation of silicon fiber using an argon-ion laser. The period of the grating thus formed was limited by the wavelength of the laser used, and the grating reflected light only in the narrow spectral region around this wavelength. An intense investigation of these gratings occurred in 1989. The greatest development of fiber gratings was recorded in the 1990s when the physical mechanism of photosensitivity was fully understood, and new methods for making gratings were developed. Since 1995, fiber gratings have been commercially available, and since 1997 have become a standard component of optical fiber connections.

### 2.2. Principle of Operation of FBG Sensors

**Figure 4:**
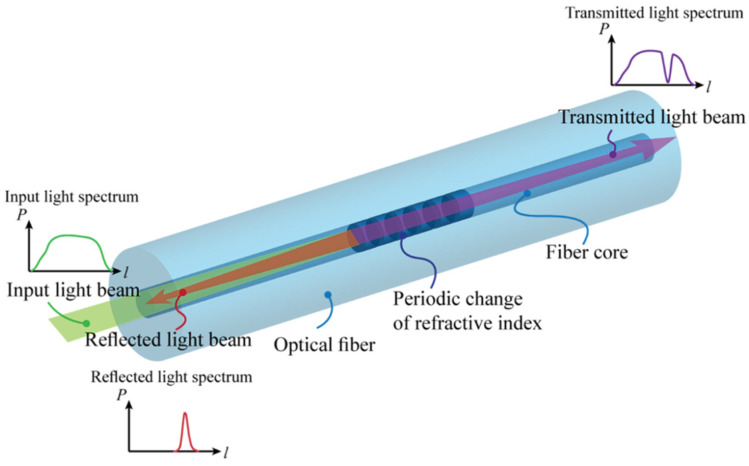
Principle of function of FBG sensors in spectral area as a part of optical fiber sensor [23,24].

The principle behind the FBG sensors is based on Fresnel diffraction (Figure 4). At the interface of two mediums with different refractive indices, the propagating optical rays can refract or reflect [19,20]. FBGs serve as reflectors of the light for specific (required) wavelengths to ensure that phase adaptation conditions are met. Other (unfavorable) wavelengths are only slightly influenced by the Bragg grating. The period of the grating Λ can be related to the Bragg wavelength *λ_B_* in this manner [21,22] where *n_eff_* is the effective core index of refraction

$$\lambda_{B}=2n_{eff}\Lambda .$$

The Bragg wavelength is a function of the period of the grating, which is why FBGs can be manufactured for various wavelengths that then allow the various FBGs to reflect unique and required wavelengths of radiation.

Changes in strain and temperature influence both parameters of FBG, namely the effective refractive index and the period of the FBG, which result in the shift of the reflected wavelength (Figure 4). This shift is due to the mechanical strain and temperature changes and can be related with the following equation [19,20]

$$\Delta \varepsilon =\frac{1}{A}\frac{\lambda_{act,strain}-\lambda_{0,inst,strain}}{\lambda_{0,inst,strain}}-\frac{B}{A}\left(T_{act}-T_{0,inst}\right),$$

where:
Δεis strain(µε),λ*_act_*_,*strain*_is actual wavelength(nm),λ_0,*inst*,*strain*_is initial wavelength(nm),*T_act_*is current ambient temperature(°C),*T*_0__,*inst*_is initial ambient temperature(°C),*A*is the strain coefficient (7.7154439915 × 10^−7^)*(µε^−1^),*B*is the thermal coefficient (5.6294839498 × 10^−6^)*(°C^−1^)*/These calibration constants are given by the manufacturer of FBGs and temperature changes were directly measured.

**Figure 5:**
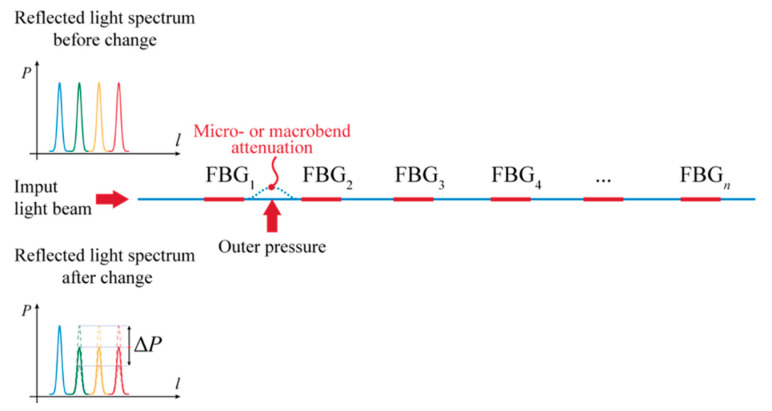
Principal representation of a directly distributed optical fiber sensor and its functioning.

From the point of view of the possible various configurations of the sensor based on FBG, several scenarios are available as to how such a sensor can function. A direct fiber sensor can be considered in the case when a reflected (damped) wavelength from each FBG is influenced by a parameter change of the optical fiber at a certain place before the FBG sensor, caused by bending for instance (Figure 5). Increased damping will affect the following reflected wavelengths, which will allow localization of the defect with some accuracy. By default, it is possible to determine the size of the deformation force with some precision. Such a (constructively easier) sensor can serve, for example, for detecting macroforms.

**Figure 6:**
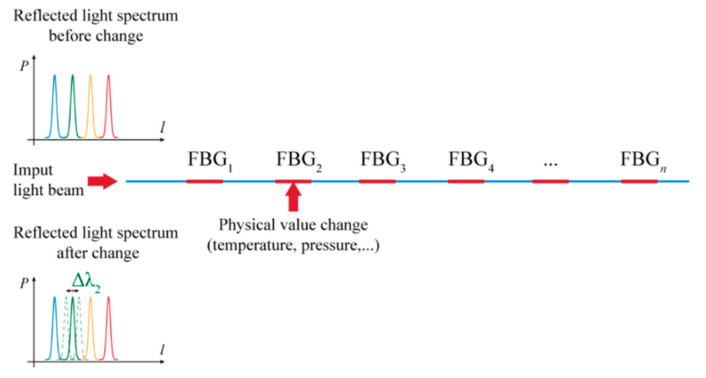
Principal representation of the indirectly distributed fiber sensor and its functioning.

**Figure 7:**
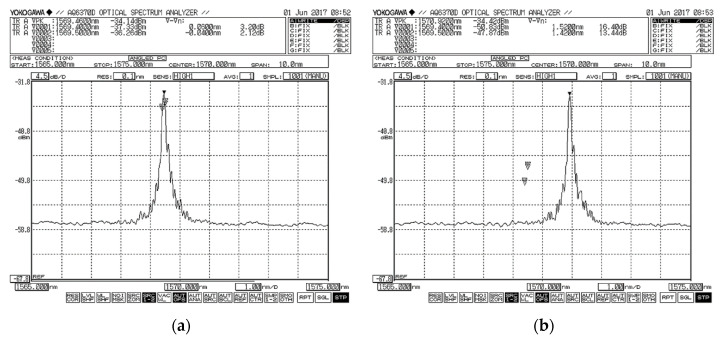
Recording of strain process of FBG sensor (**a**), change in light wavelength due to mechanical strain (**b**).

The indirect form of the fiber sensor was used in our case. This is an alternative in which the physical quantity acts directly on the FBG (Figure 6), causing a change in the grid period to induce a change in the Bragg wavelength. This allows both to detect the change in physical quantity and to locate it (position of the FBG could be known). The advantage is the possibility of reading the physical values at high accuracy [23,24]. A sample record of FBG sensor light wavelength change due to mechanical strain is on Figure 7.

## 3. Physical Model

**Figure 8:**
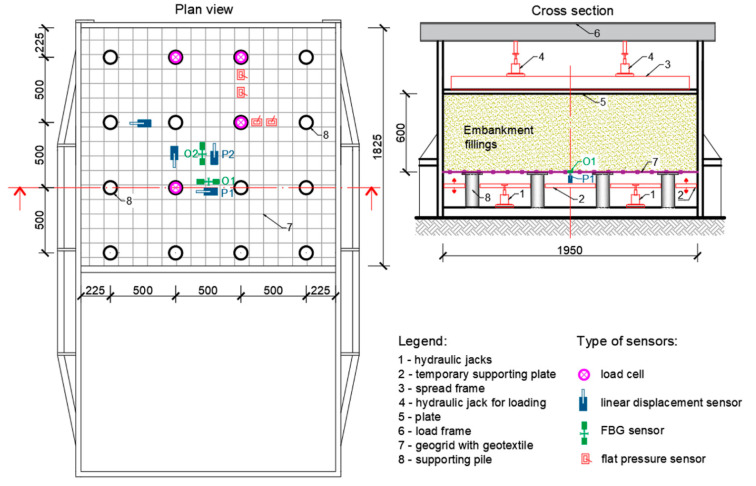
Scheme of the physical model of the piled embankment (units mm) with used sensors.

A large-scale physical model was built within project VEGA 1/0275/16 “Optimization design of sleeper subgrade due to non-traffic load aspect” [25,26] in cooperation with the Department of Railway Engineering and Track Management to monitor the construction of a piled embankment. In addition to other parameters, the axial strain of the geogrid in the reinforced base of the physical model was measured (Figure 8). The reinforced base layer (named basal reinforcement of embankment) was built from geotextile and compacted layers of crushed stones, reinforced at the bottom by a biaxial extruded geogrid.

Nowadays, physical model creation is not so popular due to the many difficulties which the research team had to overcome. These problems are related to the limit in the size of model structure, boundary conditions, and specific material properties in the scaled model and reinforcement. All of these parameters must be in appropriate relations to the real structure [27].

**Figure 9:**
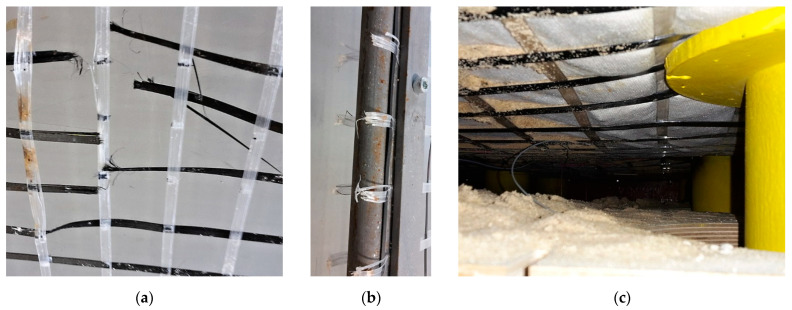
Geogrid breakage after overload (**a**), fixation problem on the edges (**b**), detail of pile support and basal reinforcement layer reinforced by geogrid and geotextile ready for testing (**c**).

First, the sensor fixation on the rigid geogrid in the basal layer upon piles must be resolved as the first attempts of model load created damage on the reinforcement strips of geosynthetic material. The geogrid ribs tore due to the inappropriate fixation of the FBG sensors, Figure 9a, where glue reacted badly with the synthetic material of the strips.

Second, to simulate the continuity of geogrid reinforcement in a real structure, a rectangular steel frame was used to prestress the geogrid by fixing it on the outer circumference. Additionally, the special friction steel strips were developed (Figure 9b) to eliminate overloading of the smoothed geogrid strips in places of fixation. After many attempts, the physical model was ready for the first loading stage measurements, Figure 9c.

**Figure 10:**
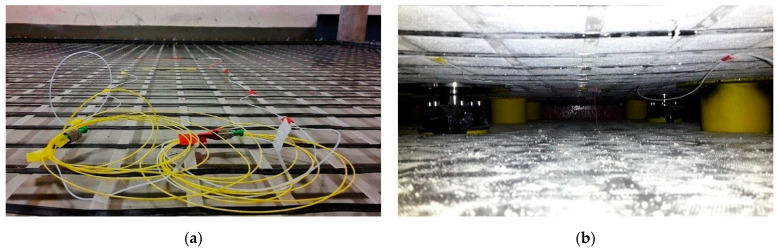
Geogrid with installed FBG sensors (**a**), view of installed sensors in physical model (**b**).

According to the material composition of the geogrid, the proper glue for the fixation of FBG sensors has to be used as every unsuccessful effort may result in a sensor loss, which is not cheap. Figure 10a shows the installed sensors on the geogrid prestressed by fixation in a steel frame before gluing. The view of the geogrid with FBG sensors during a measurement is shown in Figure 10b.

FBG sensors measured the strain elongation of the geogrid with high sensitivity and at positions (O1 and O2), and data were carried out using an optical spectral analyzer, see Figure 7. Another observed parameter was the vertical deformation (deflection) of the geogrid, which was also measured in two places (P1 and P2). For the purposes of monitoring this parameter, linear displacement sensors type TR 50 were selected. Data were collected by Spider apparatus evaluated by ABM software.

Four load cells installed on pile heads were used for measuring the distribution of the active vertical load and flat pressure sensors were also used to measure the load between supporting piles (Figure 8).

Each test started by running down the supporting plate. The supporting plate in the physical model had two functions. The first function was to allow compaction of the embankment in the process of its construction. The second function was the possibility of its vertical shift (running down below the level of the reinforced base) in order to simulate a complete loss of the bearing capacity of the subsoil. Lowering the support plate allowed us to analyze the embankment without the support of the subsoil. At this moment, the entire weight of the embankment began to act on the reinforced base and the stress in section B (13 kPa) and force on section A (3.29 kN/pile) was measured (Figure 1). This step was followed by extra loading of the load cylinders. The load phase consisted of the following six load stages: 0 → 50 → 100 → 150 → 200 → 250 → 300 kN. At each stage, a load with a duration of five minutes was applied to the embankment surface. This load was applied by four hydraulic cylinders, a spread frame, and spread plate with the dimensions of 1.8 × 1.8 m [27].

## 4. Results and Comparison

Three sets of axial strain (elongation) measurements were performed, with each set using a geogrid with different axial strength (20, 40, and 60 kN·m^−1^). In each set, the axial strain of the geogrid was measured at two locations (O1, O2) by FBG sensors with a wavelength of 1560 nm and 1580 nm [27]. Wavelengths were monitored by using an optical spectral analyzer OSA203B with spectral accuracy of ±2 ppm (parts per million, i.e., 2/106, 1.56 μm and 1.58 μm, respectively).

**Figure 11:**
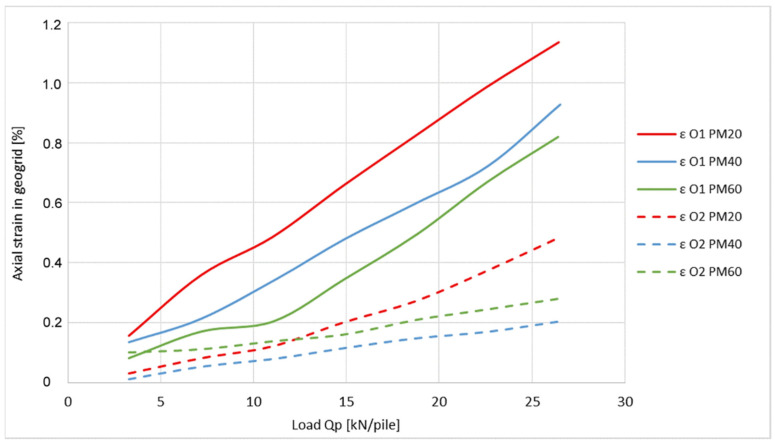
Comparison of the axial strains of the geogrid in locations O1 and O2.

Figure 11 shows the comparison of axial strain values from two different places on the geogrid of the physical model and with application of different loading on one pile.

For the purpose of proving the theory of the distribution of strain in the geogrid between piles, for measured data reliability, the following graphs displayed were compared with the outputs from the numerical modelling (PLAXIS20/40/60). Numerical modelling was carried out using the input parameters of the physical model and performed by the software PLAXIS 3D Tunnel 2.4.

**Figure 12:**
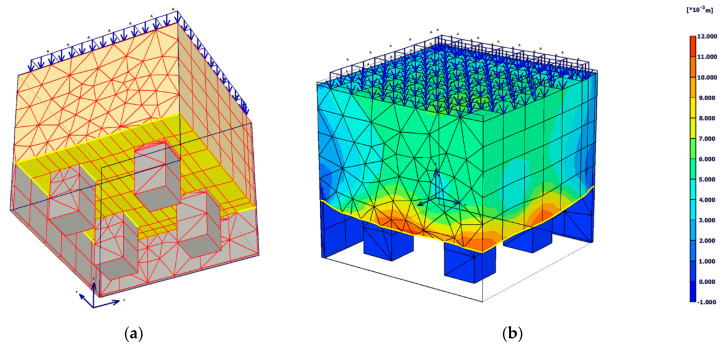
A quarter symmetrical 3D model view from bottom (**a**); total deformations of model after final stage of load 93 kPa at the top (**b**).

Since the physical model was designed symmetrically in both axes, the numerical model could be constructed as a quarter (Figure 12a). This brought benefits in the form of shortening the computational time, reducing the complexity of the numerical analysis on the hardware, and, at the same time, providing a better overview of the course of the monitored stresses and deformations in the investigated places.

The numerical computational models were optimized based on realized measurements in the physical models in the following steps:

A simple 2D PLAXIS model was first used in cross-section, which was not precise enough, therefore, the quarter axisymmetric 3D model was created, the setting of material properties of the Hardening Soil model, such as the unit weight of crushed aggregate (as embankment fillings), based on a direct test in the model, or laboratory tests. The geogrid stress–strain curve (verified stiffness) was proved during a calibration procedure, gradual optimization of the model by changing the following parameters: mesh size of full 3D model, interface values on sidewalls (boundary conditions), geogrid friction interface, and value of effective cohesion of embankment filling material.

Discretization of the continuum was performed using the function of automatic creation of a triangular network of 15 nodes elements in the 2D plane with subsequent extension to a three-dimensional network. An example of received total displacements in the 3D model of piled embankment after uniform loading at the top with the value of 93 kPa is presented in Figure 12b.

For the numerical models of the piled embankment, the Hardening Soil material model was used [27]. Mohr–Coulomb shear strength parameters of the compacted crushed aggregate were tested in the direct shear box apparatus with a size of 300 × 300 mm. The geogrid was modeled by using an elastoplastic geogrid structural element with stiffness module in a longitudinal direction J_1%_ [kN·m^−1^]. For the geogrid of 20 kN·m^−1^ tensile strength was 615 kN·m^−1^; for 40 kN·m^−1^ was 1230 kN·m^−1^, and for 60 kN·m^−1^ was 1485 kN·m^−1^.

To simulate the sidewall friction of the model, interface elements were implemented. The parameters of used materials are described in Table 1.

**Figure 13:**
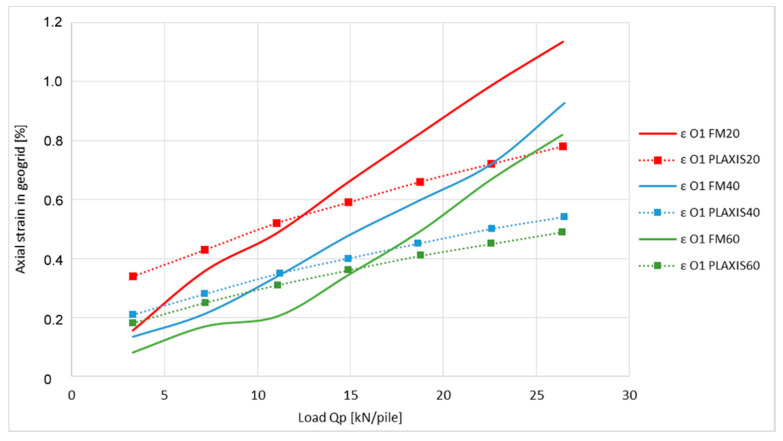
Comparison of axial strain of geogrid at place O1.

**Figure 14:**
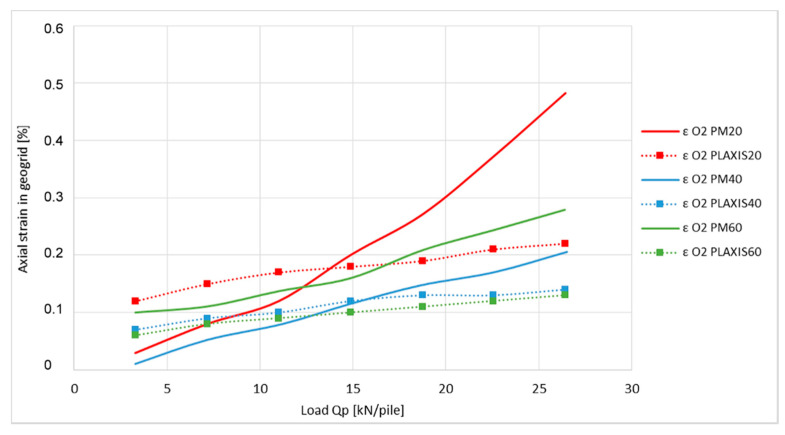
Comparison of axial strain of geogrid at place O2.

The measured values of axial strain in the places O1 and O2 obtained from the physical model (PM 20/40/60) are compared with numerical modelling results shown in Figure 13 and Figure 14.

Graphs show the outputs of three different measurements from the physical model as well as outputs of the three numerical simulations. In both of the cases, outputs are from measurements which are related to model tests where the strength of the geogrid used was 20, 40, and 60 kN·m^−1^.

As shown on the graph of the axial strain at position O1 (Figure 13), the application of the optical fiber for the purposes of determining the axial geogrid strain indicates that the outputs are in relatively good correlation with the numerical modelling. The variation in numerical analysis results can be seen mainly in the area of the last third of the measured spectrum where their reached values are 1.46 to 1.67 times of the measured value. The graph of axial strain at position O2 (Figure 14) shows larger differences, which are 1.47 to 2.2 times of the measured value.

**Figure 15:**
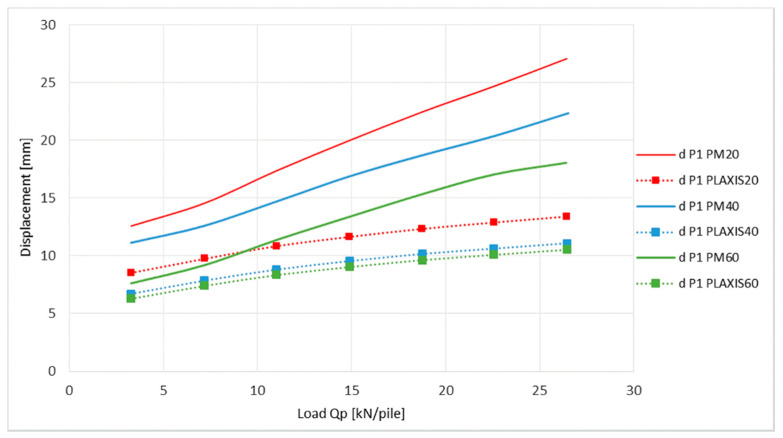
Comparison of vertical deflection of geogrid at place P1.

**Figure 16:**
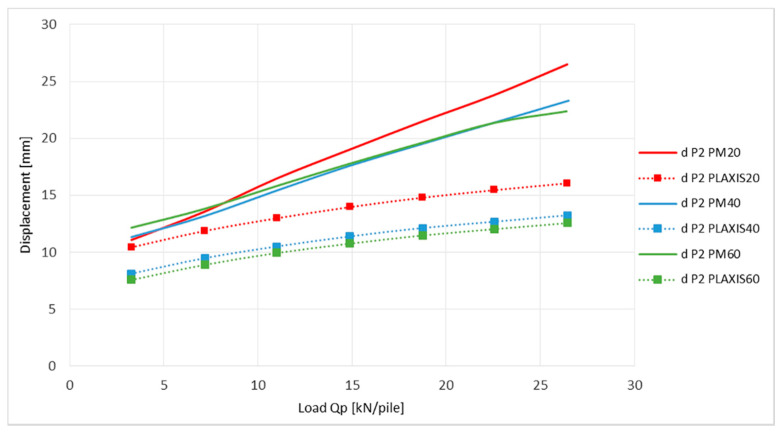
Comparison of vertical deflection of geogrid at place P2.

The axial strain differences of the geogrids, displayed in Figure 13 and Figure 14, can be explained by the shapes of the graphs in Figure 15 and Figure 16.

The graphs show the vertical deflection of the geogrid at the point of measurement of the axial strain (P1, P2). The values of deflection obtained from the numerical analysis in the end of the load stage were 1.7 to 2 times smaller for position P1 and 1.64 to 1.78 times smaller for position P2 according to measured deflection in the physical model. These differences suggest an imperfect transfer of load into the area between pile heads in the PLAXIS program, which is more probable than the inaccuracy of the FBG sensors. This condition is then generated by a smaller strain of the geogrid described in the previous chart.

Another comparison of the measurements can be introduced by comparing O1 (strain) vs. P1 (deflection) and O2 vs. P2 but indirectly. Generally, these deformations measured on the geogrid are different but dependent; this means that when axial strain *ε* is present then vertical deflection *d* also appears on the unsupported part of the geogrid. The most used theory for describing vertical deflection of the geogrid is the “cable theory” [27] where one important component is vertical load.

In our case, the theory is described as such but the reality is different due to the unequal distribution of vertical load on the horizontally installed geogrid on pile heads. The distribution depends on the interaction of the filling material with the geogrid, geometrical parameters of support, shear strength properties, and height of unreinforced filling materials. Naturally in this type of soil structure, the arches shape of a half-sphere is created (Figure 1) over an unsupported area of basal reinforcement. This phenomenon can be visible in both physical models and numerical models. For the calculation of tensile force acting in the geogrid, many authors have proposed different load schemes, such as an inverse triangle model, parabolic shape, normal triangle load scheme, or equal load. This depends strongly on the interaction between the geogrid and the filling material and the number of reinforcing geogrids [2,3,4] in the basal reinforcement layer. There was no measured load at places O1/P1 and O2/P2, therefore, a comparison of strain at O1; resp. O2 with calculated strain due to the measured deflection at P1; resp. P2 was not provided.

It must be stated that measured values O1 and O2 and P1 and P2 cannot be compared because they are in different positions and a different load is present. Point O1 is placed at the middle point between two piles as opposed to O2 which is positioned in the center of four piles (Figure 8).

## 5. Discussion and Conclusions

The presented innovative use of FBG sensors cannot miss the important target of research. Numerical computational models are common and popular in engineering applications but the reliability of results strongly depends on the skills and knowledge of the user, and the discretization of FEM code [1]. Therefore, comparison and validation with physical models or real measurements on built structures are necessary [7,24].

Nowadays, it is easy to have many trials of numerical models’ outputs, e.g., PLAXIS implemented the Python batch calculation procedure for different input parameters resulting in a lot of data. However, in our case, the numerical model was optimized in variable parameters in order to receive similar vertical deflection (sensors O1, O2) or axial strain (sensors P1, P2) of the geogrid according to the applied load. The estimation procedure by changing the parameter was used separately and the final refinement was conducted with less influenced parameter. This means that the gradual approach method was used. In the continuation of this work, such a type of optimization method could be a topic for another research.

FBG sensor applications are growing in the area of structural health monitoring where buildings, bridges, tunnels, dams, slopes, or piled embankments on transport structures are endangered by geohazards, or when it is important to monitor continuous changes during their lifetime [22,23]. This is due to the several advantages, the reason for which FBG sensors are increasingly used in any kind of monitoring. The main advantages are their small geometric dimensions, which do not disturb the surrounding interaction with the environment, their excellent stability over time, and easy installation. In the case of the monitoring of geotechnical structures, such as micropiles, driven piles, composite piles, or prefabricated concrete piles, it is very common to mill a groove in the surface and fit FBG packaging directly onto the monitored element, while for structures such as steel bars, anchors, or bored piles, FBG sensors are installed in various types of small pipes [12].

The implementation of optical fibers directly on the geosynthetic reinforcement for the purposes of determining the axial strain seems to be a reliable and accurate tool of geotechnical monitoring of reinforced soil structures (RSS) and their limit states where the most crucial factor is the tensile force of geosynthetic reinforcement [7,9,28,29]. For this type of implementation, a standard diameter of 125 μm of fiber is recommended, which is common for communication fibers. Depending on the environment of installation and the purpose of measurements, FBG sensors must be protected by appropriate coatings. In our case, standard transparent adhesive tape was used, which was enough because there was no contact with filling material (installation on the bottom side of the geogrid strip). During the construction of the physical model and measurements after loading, there was no case of lost connectivity or sensor damage. There has been one experience of a research team [30] with the installation of FBG into the asphalt pavement layer where special Kevlar coatings were efficient. There are many applications in geotechnical monitoring where fibers are installed in plastic tubes, or metal or composite plates [24]. This can be simply prefabricated by the producer of fibers according to order.

Reliable information about displacements is very important for the long term assessment of reinforced soil structures, or for other structures as well as for the assessment in the course of trial loading of piles below the RSS [31] or the structure itself [32], or for the continuous changes monitoring in the surrounding rock environment. The sensitivity of FBG sensors is enough to measure the soil consolidation at an aquifer where installed vertical Teflon tubes are able to measure vertical strain due to changes in the soil skeleton. Changes are recorded during pumping water from wells even at a radius of 350 m distance from the pumping source [11]. Several options of distributed FBG sensors packaged for monitoring and investigation of the mechanism of soil subsidence, and the evaluation of soil compression deformation potential have also been introduced [33].

Performed test results showed reliable measurements, especially for the lower and the middle area of the measured strain when numerical analyses confirmed the results, which were on the safe side. On the other hand, in the case of larger strain, the tendency is to underestimate these parameters at numerical analysis calculated with PLAXIS software. For a more reliable determination of axial strain by optical fibers, other tests are recommended, focusing on behavior in larger strains, as well as on the area of the technology of installation of FBG sensors. Because different synthetic materials are used for the production of geogrids (polyester, polypropylene, polyamide, polyethylene), the fixation of FBG sensors requires some practical training and testing. However, the successful application of optical sensors installed on geogrids was also confirmed by similar research [23,34]. If conditions require axial strains of the geosynthetic reinforcement higher than 2.5%, it will be necessary to look for an alternative to the conventional optical fiber [6,31,32].

A very important benefit of the optical fiber is, among other things, their capability in the area of dynamic analyses [35,36]. These measurements require a 2 kHz data collection device, and devices with frequencies up to 11 kHz can be used. An obstacle to the greater expansion of FBG sensors into practical, in situ implementations is their price as well as the price of the measuring equipment; forthcoming research must also resolve the reliability and durability of installed sensors.

The construction and verification of physical model tests, such as a scaled model of a piled embankment structure, requires a lot of preparation and partial testing, especially for sensor type selection and calibration. Using FBG sensors for the measurement of the axial strain of the geogrid was successfully proved; the next research activities are to continue with a laboratory model of a piled embankment with simulated partial support of the subsoil.

## Figures and Tables

**Table 1:** Material characteristics of the used numerical model of piled embankment [27].

Parameter/Material	Symbol	Pile	Embankment
Material model type		Linear Elastic	Hardening Soil
Permeability		nonporous	permeable
Unit weight	γ [kN·m^−3^]	-	21.0
Secant stiffness for primary loading in triaxial test	E_50_ [MPa]	210 × 10^6^	6 × 10^4^
Tangent stiffness for primary loading in oedometer test	E_oed_ [MPa]	-	4 × 10^4^
Stiffness in unloading/reloading	E_ur_ [MPa]	-	18 × 10^4^
Stress dependent stiffness according to a power law	m	-	0.75
Poisson’s ratio for elastic unloading/reloading	ν_ur_	-	0.2
Friction angle	ϕ [°]	-	50.2
Cohesion	c [kN·m^−2^]	-	1.0
Dilatancy angle	ψ [°]	-	20.0
Reference stress for stiffness	p^ref^ [kN·m^−2^]	-	100.0
Coefficient of lateral stress	K_o_^nc^	-	0.232
Failure ratio	R_inter_	1.0	1.0

## Data Availability

Not applicable.

## References

[1] Drusa M., Vlcek J. (2016). Importance of Results Obtained from Geotechnical Monitoring for Evaluation of Reinforced Soil Structure—Case Study. J. Appl. Eng. Sci..

[2] German Geotechnical Society (2012). Recommendations for Design and Analysis of Earth Structures Using Geosynthetic Reinforcements—EBGEO.

[3] Van Eekelen S., Bezuijen A., Van Tol A. (2013). An analytical model for arching in piled embankments. Geotext. Geomembr..

[4] Drusa M., Kais L., Vlček J., Mečár M. (2015). Piled Embankment Design Comparison. Civ. Environ. Eng..

[5] Bathurst R.J., Blatz J.A., Burger M.H. (2003). Performance of instrumented large-scale unreinforced and reinforced embankments loaded by a strip footing to failure. Can. Geotech. J..

[6] Van Eekelen S., Bezuijen A., Lodder H., Van Tol A. (2012). Model experiments on piled embankments. Part I. Geotext. Geomembr..

[7] Suits L.D., Sheahan T.C., Yang G., Ding J., Zhou Q., Zhang B. (2010). Field Behavior of a Geogrid Reinforced Soil Retaining Wall with a Wrap-Around Facing. Geotech. Test. J..

[8] Sun Y., Xu H., Gu P., Hu W. (2017). Application of FBG Sensing Technology in Stability Analysis of Geogrid-Reinforced Slope. Sensors.

[9] Osborne N.H., Ng C.C., Chen D.C., Tan G.H., Rudi J., Latt K.M., Ng C.W.W., Huang H.W., Liu G.B. (2009). Maximising the potential of strain gauges: A Singapore perspective. Geotechnical Aspects of Underground Construction in Soft Ground.

[10] Legge T.F.H., Swart P.L., Van Zyl G., Chtcherbakov A.A. (2006). A fibre Bragg grating stress cell for geotechnical engineering applications. Meas. Sci. Technol..

[11] Drusová S., Wagterveld R.M., Wexler A.D., Offerhaus H.L. (2019). Dynamic Consolidation Measurements in a Well Field Using Fiber Bragg Grating Sensors. Sensors.

[12] Hong C.-Y., Zhang Y.-F., Zhang M.-X., Leung L.M.G., Liu L.-Q. (2016). Application of FBG sensors for geotechnical health monitoring, a review of sensor design, implementation methods and packaging techniques. Sens. Actuators A Phys..

[13] Pei H.-F., Li C., Zhu H.-H., Wang Y.-J. (2013). Slope Stability Analysis Based on Measured Strains along Soil Nails Using FBG Sensing Technology. Math. Probl. Eng..

[14] Sun L., Li H.-N., Ren L., Jin Q. (2007). Dynamic response measurement of offshore platform model by FBG sensors. Sens. Actuators A Phys..

[15] Kadela M. Response of subsoil to cyclic load transferred by pavement. Proceedings of the GeoShanghai International Congress: Pavement Materials, Structures, and Performance.

[16] Zhou J., Sun L., Li H. (2014). Study on Dynamic Response Measurement of the Submarine Pipeline by Full-Term FBG Sensors. Sci. World J..

[17] Wang Z., Wang J., Sui Q., Liang X., Jia L., Li S., Lu S. (2015). Development and Application of Smart Geogrid Embedded with Fiber Bragg Grating Sensors. J. Sens..

[18] Hill K.O., Fujii Y., Johnson D.C., Kawasaki B.S. (1978). Photosensitivity in optical fiber waveguides: Application to reflection filter fabrication. Appl. Phys. Lett..

[19] Haus J. (2010). Optical Sensors: Basics and Applications.

[20] Yin S., Yu F.T.S., Ruffin P.B. (2008). Fiber Optics Sensors.

[21] Venghaus H. (2006). Wavelength Filters in Fiber Optics.

[22] Zhu H.-H., Shi B., Zhang C.-C. (2017). FBG-Based Monitoring of Geohazards: Current Status and Trends. Sensors.

[23] Campanella C.E., Cuccovillo A., Campanella C., Yurt A., Passaro V.M.N. (2018). Fibre Bragg Grating Based Strain Sensors: Review of Technology and Applications. Sensors.

[24] Sahota J., Gupta N., Dhawan D. (2020). Fiber Bragg grating sensors for monitoring of physical parameters: A comprehensive review. Opt. Eng..

[25] Dobes P., Izvolt L. Experimental monitoring of moisture changes in railway track structure. Proceedings of the Transcom 2015: 11th European Conference of Young Researchers and Scientists.

[26] Ižvolt L., Dobeš P., Pitoňák M. (2017). Preliminary results and conclusions from the experimental monitoring of thermal regime of railway track structure. Int. J. Transp. Dev. Integr..

[27] Kais L. (2017). Reinforced Soil Structures on Unbearable Subsoil. Ph.D. Thesis.

[28] Liehr S., Lenke P., Wendt M., Krebber K., Glötzl R., Schneider-Glötzl J., Gabino L., Krywult L. Distributed Polymer Optical Fiber Sensors in Geotextiles for Monitoring of Earthwork Structures. Proceedings of the 4th International Conference on Structural Health Monitoring on Intelligent Infrastructure.

[29] Sun Y., Cao S., Xu H., Zhou X. (2020). Application of Distributed Fiber Optic Sensing Technique to Monitor Stability of a Geogrid-Reinforced Model Slope. Int. J. Geosynth. Ground Eng..

[30] Matula M., Dubovan J., Markovic M. Sensors utilization for weighing applications of vehicles in motion. Proceedings of the ELEKTRO 2014.

[31] Muszynski Z., Rybak J. (2017). Horizontal Displacement Control in Course of Lateral Loading of a Pile in a Slope, WMCAUS 2018 symposium. IOP Conf. Ser. Mater. Sci. Eng..

[32] Hildebrand M., Rybak J. (2017). Evaluation Criteria and Results of Full Scale Testing of Bridge Abutment Made from Reinforced Soil. IOP Conf. Ser. Mater. Sci. Eng..

[33] Wu J., Jiang H., Su J., Shi B., Jiang Y., Gu K. (2015). Application of distributed fiber optic sensing technique in land subsidence monitoring. J. Civ. Struct. Health Monit..

[34] Juraszek J., Gwóźdź-Lasoń M., Logoń D. (2021). FBG Strain Monitoring of a Road Structure Reinforced with a Geosynthetic Mattress in Cases of Subsoil Deformation in Mining Activity Areas. Materials.

[35] Her S.-C., Yang C.-M. (2012). Dynamic Strain Measured by Mach-Zehnder Interferometric Optical Fiber Sensors. Sensors.

[36] Ma G.-M., Li Y.-B., Mao N.-Q., Shi C., Zhang B., Li C.-R. (2018). A Fiber Bragg Grating-Based Dynamic Tension Detection System for Overhead Transmission Line Galloping. Sensors.

